# Salvaging the Lost Pink Triangle: A Case Series of Papilla Reconstruction

**DOI:** 10.1155/2020/9735074

**Published:** 2020-01-14

**Authors:** Santhosh Shenoy B, Anahita Punj, Amitha Ramesh, Avaneendra Talwar

**Affiliations:** ^1^Department of Periodontics, A.B Shetty Memorial Institute of Dental Sciences, NITTE (Deemed-to-be) University, Deralakatte, Mangalore, Karnataka-575018, India; ^2^Army College of Dental Sciences, Jai Jawahar Nagar, Chennapur-CRPF Road, Secunderabad, 500087 Telangana, India

## Abstract

**Introduction:**

The interdental papilla may be lost or reduced in height, forming black triangles due to various reasons, which gives an unaesthetic appearance when the patient smiles. Various noninvasive and invasive techniques have been used to augment/reconstruct the interdental papilla, to reclaim the pink triangle. The most satisfactory and natural appearance can be obtained by augmenting or reconstructing the lost papilla as the pink esthetics is as important as the white esthetics.

**Cases:**

Two female patients and 1 male patient reported to the dental department with the complaint of a small black gap in the gum area between their upper front teeth since 6 months and 1 year, respectively. On examination, the interdental papillae in all three cases were classified as class 1 (Nordland and Tarnow's). The interdental papilla was augmented surgically by using three different approaches in each case.

**Result and Conclusion:**

Postoperatively and after 1-month follow-up, there was a complete fill of the interdental area by the interdental papilla normal (Nordland and Tarnow's). As a result, the black triangle was successfully converted into a natural pink triangle in all three cases.

## 1. Introduction

The interdental papilla is a component of the gingiva which is present between the proximal surfaces of the teeth occupying the cervical embrasure space which extends to fill the lingual, buccal, and occlusal pyramidal space of the interdental space [1]. The interdental papilla is triangular when observed two dimensionally and pyramidal when viewed three dimensionally. The shape, position, and presence is dependent upon the presence of underlying alveolar bone proper, the proximal contact point, and the area of the adjacent teeth [1]. This entity is also referred to as the interdental papillary house by Gonzalez et al. [2] where the dimensions of the roof, floor, and lateral walls of the interdental house play important roles in maintaining the interdental papilla.

It is needless to say that in the absence of tooth, alveolar bone proper, and adjacent tooth contact, the interdental papilla will fail to sustain its original form and result in an empty space between teeth, which is commonly referred to as a black triangle. The black triangle can result in unaesthetic appearance while smiling and can result in food impaction and phonetic problems [3]. The etiologies which can result in black triangle include aging, periodontal disease, loss of height of the alveolar bone relative to the interproximal contact, length of embrasure area, root angulations, tooth loss, faulty oral hygiene procedures, interproximal contact position, and triangular-shaped crowns [4, 5].

Once the papilla is lost due to any of the above factors, rebuilding it again and expecting regeneration is cumbersome. Papilla reconstruction or its augmentation is the most esthetically challenging procedure in perioplastic procedure [6]. A number of techniques have been developed by various authors such as Beagle [7], Azzi et al. [8], Han and Takei [9], Carnio [6], and Froum et al. [10] in an attempt to convert the black triangle into an aesthetic pink triangle. The methods include nonsurgical and surgical methods (Table 1). In many cases, surgical methods if performed correctly can give everlasting and natural results. This article attempts to observe and compare the outcome of three different procedures carried out to reconstruct the papilla.

## 2. Case Presentation

### 2.1. Case 1

**Figure 1:**
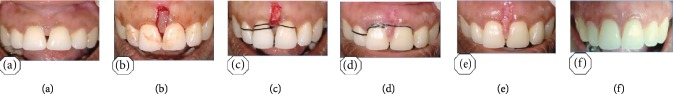
(a) Preoperative condition of the papilla between 11 and 21 with square shaped teeth, (b) interproximal and vertical incisions starting at the base of the papilla and connected by horizontal incision, (c) split thickness flap is reflected and rolled partially over to fill the black triangle area and sutured circumferentially around the adjacent teeth, (d) 10 days after operative procedure, (e) 3 weeks after suture removal, and (f) 2 years after procedure.

A 45-year-old adult female patient reported with complaint of deposits in the lower anterior teeth and a black space between the upper front teeth and the gum region since 6 months. On examination, the patient presented with class 1 papilla (Nordland and Tarnow's [11]). The patient was diagnosed with chronic papillary gingivitis [12] with papillary gingival recession type 1 [13] (Figure 1(a)). As the interdental bone was within 5 mm from the contact point, the roll technique [11, 13] was used for reconstruction of the papilla. Scaling and root planning was performed for the patient on the first visit and brushing technique was corrected. After 1 week, surgical procedure was carried using the roll technique. The interdental area was anesthetised by local infiltration using 0.2% lignocaine with 1 : 80000 epinephrine. Next, two parallel vertical incisions were placed in the interdental gingiva [12] starting from the coronal end of the papilla near the proximal contact points and was extended apically and joined by a horizontal incision to create a pouch (Figure 1(b)). A partial thickness flap was raised from the apical end and was gently advanced apically towards the contact point. The partial thickness was then rolled and folded on itself and sutured using 3-0 silk suture to keep it in the designated position. While suturing, the suture was circumferentially wound around adjacent teeth to avoid any displacement (Figure 1(c)). Periodontal dressing was given to provide mechanical protection, and the patient was instructed to take analgesics twice a day to avoid postoperative pain. Suture removal was done after 10 days (Figure 1(d)), and there was complete fill of the interdental papilla which was maintained even after 3 weeks (Figure 1(e)) and after 2 years (Figure 1(f)).

### 2.2. Case 2

**Figure 2:**
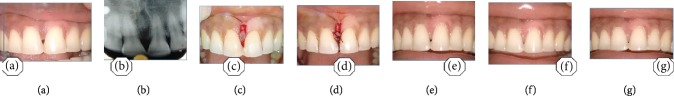
(a) Preoperative condition of the papilla between 11 and 21 with triangular-shaped teeth, (b) intraoperative periapical radiograph showing more than 5 mm of vertical distance between the contact point and the bone crest, (c) interproximal and vertical incisions starting at the base of the papilla and connected by horizontal incision, (d) split thickness flap is reflected and rolled partially over to fill the black triangle area and sutured over composite stops, (e) 10 days after operative procedure, (f) 2 months after procedure, and (g) 2.5 years after procedure.

A 30-year-old adult female patient reported with complaint of a black space between the upper front teeth and the gum region since 6 months. The patient gave a past history of orthodontic treatment. On examination, the patient presented with class 1 papilla [11]. The patient was diagnosed with chronic papillary gingivitis with papillary gingival recession type 1 [12, 14] (Figure 2(a)). Scaling and root planning was performed for the patient on the first visit. After 1 week, as the interdental bone was slightly more than 5 mm from the contact point (Figure 2(b)), the contact point was moved coronally by building the proximal contours with composite restoration to allow creeping attachment to occur postsurgery (Figure 2(c)). Next, surgical procedure was carried using the roll technique in the same manner as in case 1. While suturing, composite stops were used to suspend the suture and avoid any displacement (Figures 2(c) and 2(d)). Periodontal dressing was given to provide mechanical protection, and the patient was instructed to take analgesics twice a day to avoid postoperative pain. Suture removal was done after 10 days (Figure 2(e)), and there was almost complete fill of the interdental papilla, and after 2 months (Figure 2(f)), the interdental papilla completely filled the interdental area. The papilla was maintained in a stable position even after 6 months postprocedure (Figure 2(g)). Unfortunately, the patient was lost to follow-up as she moved to some other city.

### 2.3. Case 3

**Figure 3:**
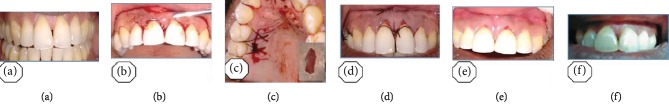
(a) Preoperative condition of the papilla between 11 and 21 with buccal gingival recession, (b) pouch and tunnel created in the interdental papillary area extending to 11 and 21 distally using microsurgical instruments, (c) connective tissue graft (inset) harvested from the palate and recipient site sutured, (d) connective tissue graft placed inside the tunnel using lasso suturing and positioned using sling sutures, (e) 1 month after operative procedure, and (f) 2 years after the procedure.

A 43-year-old adult male patient reported with complaint of a black space between the upper front teeth and the gum region since 6 months. On examination, the patient presented with class 1 gingival recession and class 1 papilla [11]. The patient was diagnosed as chronic generalised marginal gingivitis with papillary gingival recession type 2 with 11 and 21 (Figure 3(a)) [12, 14]. As the interdental bone was more than 5 mm from the contact point, the pouch and tunnel technique with interposed connective tissue graft using microsurgical instruments was planned. Scaling and root planning was performed for the patient on the first visit. After 1 week, surgical procedure was carried by anesthetising the interdental area by local infiltration using 0.2% lignocaine with 1 : 80000 epinephrine. Next, under magnification of 2.5x, a sulcular incision was given around the facial and proximal surfaces; next, using microsurgical tunnelling instruments, a subperiosteal pouch was created by raising a partial thickness flap using sharp dissection (Figure 3(b)). The pouch was extended towards the interdental gingiva, detaching it from the palatal aspect partially. The tunnel was extended beyond the mucogingival junction to allow coronal movement of the interdental gingival unit. Once the interdental gingiva was coronally advanced passively, the amount of connective tissue graft required was estimated by using a tin foil and measuring the amount of space present in the subperiosteal tunnel. The tin foil was used as a guide to harvest the connective tissue graft from the hard palate using the trap door technique. After harvesting the connective tissue graft, the donor site was sutured using 3-0 silk suture with the horizontal mattress suturing technique (Figure 3(c)). The connective tissue graft was then tied at one end with 5-0 resorbable suture as a lasso suture and was inserted into the tunnel created by pulling the suture and tying it to stabilize its position [15]. Similarly, the other end of graft was also stabilized by suturing. Next, 3-0 silk suture was allowed to pass through the labial interdental gingiva, connective tissue graft, and palatal gingiva to bind the graft together and was tied as a sling suture with a composite stop in the middle of the two incisors (Figure 3(d)). Periodontal dressing (Coe-Pak) was given to provide mechanical protection, and the patient was instructed to take analgesics twice a day to avoid postoperative pain. Suture removal was done after 10 days, and there was almost complete fill of the interdental papilla, and after 1 month (Figure 3(e)), the interdental papilla completely filled the interdental area. After approximately 2 years, the interdental papillary fill showed a relapse of about 0.5 mm (Figure 3(f)). In all the three cases, the papilla presence score decreased, quantifying the gain in the papilla as shown in Table 2.

## 3. Discussion

Treating papillary recession is of utmost importance as the evidence suggests that treated gingival recession defects are unlikely to have an increase in recession depth versus untreated recession defects [16, 17]. Over the years, a number of authors have advocated methods to treat the aesthetically challenging black triangle. Nonsurgical methods such as correction of faulty tooth habits, repeated curettage of papilla every 15 days for 3 months [18], reshaping of contacts and contours using restorations or prosthesis [19], and orthodontic repositioning [20, 21] have been used in mild forms of papillary loss. When the papilla cannot be augmented by nonsurgical methods, surgical techniques become an indispensable part of the treatment plan [22]. The techniques include papilla preservation flap [23], modified papilla preservation [24], and simple papilla preservation flap [25] to prevent inadvertent papillary loss as a result of flap surgery. The other papilla reconstruction techniques include the pedicle flap using the roll technique, semilunar coronally positioned flap, envelope flap which is augmented by use of connective tissue graft [26], hyaluronic acid [27]. Platelet-rich fibrin (PRF) [5], and autogenous graft [28] especially in case the interdental bony crest and contact point are more than 5 mm apart. The use of microsurgical approaches has also been used for papilla reconstruction which cause lesser trauma and better healing outcomes [4]. Similar techniques have been used to create interimplant papilla which include the use of connective tissue graft [6], PRF [29], autogenous bone, metal pins [30], modified instrument design by Froum et al. [10], and papilla amplification flap [31].

In the first case, the patient had chronic localised gingivitis, faulty brushing of teeth, and advanced age which could have resulted in the papilla loss. Since the radiograph showed that the interdental bone crest was within 5 mm from the contact point of 11 and 21, according to Tarnow et al.'s rule [32], it was expected that a simple surgical procedure like Beagle's modified roll technique will result in postoperative papillary fill. The results of this case were similar to those obtained by Kapoor et al. [33]. In the second case, the possible etiologic factors for papillary loss were past orthodontic treatment and triangular shape of the crown. After radiographic examination, as the distance was more than 5 mm, the contact point was moved coronally by building the mesial proximal contacts with composite restoration to allow creeping movement of the gingiva. This result is supported by the fact that decreasing the height of the interdental housing can allow the papilla to maintain its presence [2]. The vertical incisions given in the roll technique used in the two cases in this report are similar to the modification given by Checchi and Schonfeld [13]. In the third case, the possible etiologies included faulty brushing of teeth and past periodontal disease. As in this case, amount of loss was greater and the gingival phenotype was thin; use of connective tissue graft to augment the papilla was used, and to avoid any inadvertent trauma during the surgical procedure, microsurgical instruments were used under 2.5x magnification. The results of this case are in agreement with cases by Carnio [6] and Azzi et al. [8] and a study done by Kaushik et al. [34]. Katsuhiko [35] also carried out papilla reconstruction using connective tissue graft using a microscope. In the third case, there was a relapse of 0.5 mm, which probably occurred due to the physiological process of aging. Montevecchi et al. [22] in their study have mentioned that papilla receded more as the age of the patient advanced. They have also inferred that less than 1 crown width/crown length makes the crown triangular and holds true for cases 1 and 3, while 1 represents square-shaped teeth which is observed in case 1. The cases included in this manuscript are comparing only the central incisor papilla and do not include the lateral incisor papilla to avoid any bias in the results as the critical horizontal and vertical distances for the central and lateral incisors which could support the 90-100% of papillary fill varies [22]. In our present cases, the postoperative discomfort was least in cases 1 and 2 as it did not involve a second surgical site. In case 3, the patient did not complain of any pain 2 days after the procedure owing to his high pain threshold and timely intake of analgesics and adherence to postoperative care. Additionally, there were no complications related to the harvesting of connective tissue graft in the present case which supports the use of grafts if needed. This fact is supported by a study done by Harris et al. [36] where the complication rate such as pain, infection, bleeding, and swelling with the connective tissue grafting procedure was low and was clinically acceptable. Shruthi et al. [37] have compared the Azzi et al. [8] and Han and Takei [9] techniques and found them equally successful. In the present paper, although we have tried to classify the papilla based on different indices to check the postoperative results, an index, called the interdental pressure index, could not be used as it required additional armamentarium and was discovered during the literature review after the procedures were carried out. The index takes into account the inflammatory status of the interdental papilla by evaluating the pressure applied by a modified dynamometer [38]. The number of cases in this report is too small to establish a proper guideline for selection of the best technique for papilla reconstruction.

The present report compares three different techniques for papilla reconstruction which were selected based upon careful diagnosis and treatment planning. An attempt has been made to achieve positive results using simple to moderate techniques for papilla reconstruction which are reproducible and amenable to the patients. On comparing the three cases, Beagle's roll technique with Checchi's modification is simpler and less painful for the patients, but it has its inherent limitations due to which connective tissue graft is used in cases where the bone support and the availability of tissue is limited. The key message is not to promote a particular technique but to diagnose the papillary loss correctly and use the predictable techniques and avoid unnecessary complex procedures if required to suit patients from all walks of life.

## Figures and Tables

**Table 1:** 

S. no.	Year	Author	Technique
1	1956	Kromer [39]	First report of papilla preservation procedure
2	1959	Cohen [40]	Described the interdental papilla
3	1963	Ochsenbein and Bohannon [41]	Palatal approach procedure in which incisions are given from the palatal aspect
4	1967	Frisch et al. [42]	Curtain procedure, which maintains the labial gingiva and lingual and interproximal 2/3rd part is released
5	1973	App [43]	Intact papilla flap: interdental gingiva is retained in the buccal flap
6	1974	Ramfjord and Nissle [44]	Use of submarginal incision to preserve papilla during flap surgery
7	1985	Shapiro [18]	Used periodic curettage of papilla to increase size of papilla
8	1985	Evian et al. [45]	Modified the intact papilla technique
9	1985	Takei et al. [23]	Papilla preservation technique and use of palatal semilunar incision 3 mm apical to gingival margin
10	1988	Checchi and Schonfeld [13]	Modified Evian and Takei's technique and suggested to give buccal incision and coronally advance it to cover graft in the palatal area. Lingual incision was to be given when maximum depth of defect was in labial aspect
11	1992	Beagle [7]	Combines principles of Abrams' roll technique for ridge augmentation with augmentation of Evian's papilla preservation technique. One treatment option for this problem has been to create on acrylic resin mask to mimic the appearance of healthy gingiva
12	1995	Cortellini et al. [24]	Modified papilla preservation technique: here the buccal flap is freed and straight interproximal incisions are given
13	1998	Azzi et al. [8]	Connective tissue from retro molar area is placed in interdental area after raising split thickness flap
14	1999	Cortellini et al. [25]	Simplified papilla preservation technique by giving an oblique incision in the interdental papilla in cases where the interdental distance is less than 2 mm
15	2001	Nemcovsky [26]	Interproximal augmentation of papilla using a new instrument and advancing the papillary flap plus placing connective tissue graft
16	2004	Carnio [6]	Papilla reconstruction using subepithelial connective tissue graft
17	2004 and 2005	Cardaropoli et al. [20, 21]	Use of orthodontics to correct lateral walls of the interdental papilla housing
18	2006	Kotschy and Laky [46]	Bone grafting done in interdental areas to raise the floor of interdental papilla housing
19	2006	Zucchelli and De Sanctis [31]	Papilla amplification flap: giving parabola incisions adjacent to the papilla to be amplified
20	2007	McGuire and Scheyer [47]	Augmentation of papilla by injecting autologous fibroblasts
21	2008	Nordland and Sandhu [15]	Microsurgical technique to augment papilla
22	2009	Akiyama [48]	Papilla reconstruction done using a microscope
23	2016	Froum et al. [10]	New surgical technique for interimplant papilla reconstruction by giving oblique incisions in the vestibular area labially and palatally and creating a tunnel using translingual curette to advance the papillary unit

The table shows the baseline (preoperative) and final (postoperative) changes in classification and gain in soft tissue level in the gingiva.

**Table 2:** 

Parameters	Case 1		Case 2		Case 3	
Timeline	Pre-op	Post-op	Pre-op	Post-op	Pre-op	Post-op
Classification [11]	1	0	1	0	1	0
Recession type [14]	Papillary gingival recession type 1	No recession	Papillary gingival recession type 1	No recession	Papillary gingival recession type 2	Buccal gingival recession
Distance from bone crest to contact point	5 mm	5 mm	6 mm	6 mm	6 mm	6 mm
Distance from contact point to gingival margin	3 mm	0 mm	4 mm	0.5 mm	4 mm	0.5 mm
Papilla presence index [49]	2	1	2	1	3r	1r

The table shows the baseline (preoperative) and final (postoperative) changes in classification and gain in soft tissue level in gingiva.
